# Herbert Tabor (1918‐2020)*

**DOI:** 10.1096/fba.2020-00106

**Published:** 2021-01-09

**Authors:** Judith S. Bond, Ralph A. Bradshaw

**Affiliations:** ^1^ University of North Carolina Chapel Hill NC USA; ^2^ University of California Irvine CA USA

Dr. Herbert Tabor, biochemist, renowned authority on the synthesis and function of polyamines, and Chief Editor extraordinaire for the Journal of Biological Chemistry (JBC) for approximately 50 years, died August 20^th^ at his home on the National Institutes Health campus in Bethesda Maryland. He was preceded in death by his wife Celia Tabor, a physician scientist and his professional colleague, who died in 2012. He is survived by their four children, Edward, Marilyn, Richard and Stanley, 10 grandchildren and 6 great‐grandchildren (Figure 1).

**FIGURE 1:**
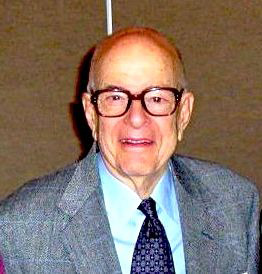
Herbert Tabor (photo by Gaylen Bradley)

Herb was born in New York City, was educated in the public schools and graduated from Townsend Harris High School in 1933. He attended City College for two years, then transferred to Harvard College and received his bachelor’s degree and MD there, in 1937 and 1941, respectively. During his last year in medical school, he spent six months working in the laboratory of A. Baird Hastings, who was Chair of the Biological Chemistry Department at Harvard Medical School, and overlapped with such well‐known figures as Birgit Vennesland, Jack Buchanan, Chris Anfinsen and Oliver Lowry. His first paper in the JBC arose from his studies in Hastings’ lab. In 1942, he interned at Yale‐New Haven Hospital and spent some of his spare time in the laboratory of John Peters, who he credits with being an important influence on his thinking. He was also involved in the first major clinical trial of penicillin in the United States and the successful treatment of a case of streptococcal septicemia. After being commissioned in the U.S. Public Health Service in early 1943, he spent his first six months at sea as the sole medical officer on the Coast Guard cutter, U.S.S. Duane. One of us (RB) experienced some of his memories of this service during a birthday festschrift for Bob Hill in the summer of 1995, at the Rosario Resort on Orcas Island in Washington. Several of us were having dinner together in the resort hotel, and as we entered the restaurant Herb stopped abruptly in front of a large group of photographs of various ships and other marine subjects and then announced with a huge grin, “That was my ship.” It was indeed the Duane and we were subsequently entertained through dinner with several anecdotes of a “very young and surgically inexperienced Dr. Tabor avoiding one medical catastrophe after another” (or at least that was how he related it). He did admit he was extremely glad to get to the NIH in the fall of 1943, his next posting, and back to the laboratory.

Herb’s association with the NIH was to be for the rest of his life, totaling some 77 years, the longest term of any NIH employee. When he first arrived at the NIH, he was assigned to the laboratory of Sanford Rosenthal where his initial efforts were on electrolyte changes in burns and shock. When this war time research wound down, Tabor and Rosenthal moved into studies on the metabolism of histamine and histidine that ultimately led to the pioneering work, with his wife Celia, on polyamines, which would characterize the rest of their careers. She was also trained in medicine and joined the NIH in late 1952. They had met several years earlier in Boston and were married in 1946. Three of their children were born prior to her USPHS commission while she was at George Washington University Medical School; the fourth, a couple of years later. Much of their early studies were focused on enzymology, and this emphasis underscores his active interactions with Bernie Horecker, Leon Heppel and Arthur Kornberg, who were colleagues with him at the NIH during his first several years there. He has described with enthusiasm their regular daily luncheon discussions/seminar meetings that started in 1946, and spanned approximately 50 years, and the importance of these interactions to the development of his research career. Arthur Kornberg recalled fondly at Herb’s 90^th^ birthday celebration in 2008, that Herb became so enthusiastic and intent on the seminar presentation on one occasion that he unknowingly ate Arthur’s birthday sandwich that was adjacent to him rather than his own! Over the years, the luncheon meetings expanded to include many other notable figures in biochemical research. The combined efforts of the Tabors heralded the explosive growth of polyamine research, trained many postdoctoral fellows, and laid the foundation of this still active area of research.

A critically important part of scientific advances is the accurate review of experimental data and entry into the scientific record. It could even be argued that the publishing of science is equally important as the discoveries themselves. Although many accomplished scientists devote significant parts of their career to scientific publishing, few have had the commitment and dedication to these activities that Herb had. Herb was on the editorial board of the JBC from 1961 to 1966 and then became an Associate Editor. Bill Stein, Editor of the JBC and a Rockefeller University professor and Nobel Laureate, contracted Guillain‐Barre Syndrome in the late 1960s, and could not continue in his role as Editor. One of us (RB) remembers events at the meeting in Copenhagen in June of 1969 when Stein was stricken and spending the last night before he became ill at a reception with him at the Carlsberg Laboratory. Herb claimed, in his humble manner, that he was asked to take on the Editor responsibilities because he resided in the immediate vicinity of the American Society of Biological Chemists (ASBC) office and the business side of the Journal. More likely his ability to lead the Journal was well recognized; he assumed the role of Acting Editor in 1969 and Chief Editor in 1971 and remained in that role for the next 41 years.

The Journal, which was launched in 1905 by founding editors Christian Herter and John Abel as an independent entity, had enjoyed, in addition to Stein, five earlier editors, four of whom had been managing editors, meaning that they were responsible for both the editorial and financial aspects of journal operations. John Edsall, who took over the editorship in 1958, oversaw the transition of the business side to the “new” offices of the ASBC (which became the American Society for Biochemistry and Molecular Biology (ASBMB) several years later) so that Stein and then Herb were able to concentrate on the editorial side of things. The ASBC had assumed the responsibility for the JBC in 1919. Stein hardly got started as Editor before he was taken ill, but Herb more than made up for any lost time leading a phenomenal 45 year campaign of growth in size and prestige that included such important events as bringing the JBC on‐line, the first biomedical journal to achieve this landmark accomplishment. In his own unassuming but steadfast approach, with a rigid attention to detail, an uncompromising commitment to excellence and a devotion rarely seen, he became a legend in science publishing, and he will long be remembered as such.

Most impressively, Herb carried out all of his JBC activities on nights and weekends, while he and Celia worked in their NIH laboratory during the day, scrupulously avoiding any conflict with his government obligations. He oversaw a spectacular growth in the Journal, from 3,100 pages in 1968 to surpassing 55,000 published pages/year at one point. Likewise, the number of Associate Editors increased from 3 in 1968 to over two dozen recently, and editorial board members increased from 54 to hundreds from all over the world, illustrating that the size of his commitment was truly staggering. Herb also resisted pressure to print “hot” or “flashy” science but rather felt that all science that makes a substantial contribution to the biochemical and molecular biological literature should be published in the Journal. This attitude is the basis for the reputation of the JBC for outstanding, high quality publications that advance science and stand the test of time. Herb was also highly protective of the Journal and its assets and would become quite aggressive in shielding it when he thought the Finance Committee or the Council was contemplating using monies he considered were the Journal’s for other activities. This led, on more than one occasion, to some interesting exchanges. One such instance was the development of the online version of the Journal. Although he clearly recognized the value of such a venture, if successful, he also recognized that it could fail and end up financially crippling both the Journal and the Society. One of us (RB) was Treasurer at the time and had many of the same qualms. Witnessing his cautious and careful support of the project while at the same time not allowing it to get out of control was a perfect example of his leadership skills.

Herb’s contributions to the ASBC/ASBMB were also substantial and are in many respects underappreciated. The histories of the Society and the Journal are entirely interwoven, even though they started separately, and the combination has been a significant and powerful contributor to the advance of bioscience. Everyone involved in the governance of the Society was aware of his constant presence and thoughtful contributions at meetings of the Council, the Financial Committee, as well as the Publications Committee, which spanned his time as the Editor of the JBC. And he did it all with a fantastic sense of balance and humor. He was nearly unflappable and was a calming influence regardless of how heated discussions became. He was at his best leading JBC Associate Editor meetings, commonly and accurately referred to as “the proverbial herd of cats” and securing consensus on topics that one would swear could not be reconciled.

Herb was one of the calmest, most insightful and humblest individuals that either of us has known. He did have his moments though and felt passionately about some subjects. For example, one incident occurred several years ago and came about from a suggestion by the Publications Committee that the journal might be more appealing with a flashier name. On leaving the Publication Committee meeting where this suggestion had been made, Herb joined the ongoing meeting of the Council with the look of a man who had just had to shoot his dog. It was plainly evident that he was thoroughly distressed, and eventually related what the problem was in a private conversation. While this might not seem at first blush to be of such great concern, due to an accident of history, the Publications Committee had been originally created as a separate body within the society and was, at the time, still independent of Council and the elected officers, that is, it could make its own decisions that did not require any ratification or approval from any other part of the society. Thus, if it wanted to change the name of the JBC to something else it had the full authority to do so, and Herb was clearly afraid that it might choose to do just that. The only obvious solution was to change the society’s bylaws and end the autonomy the Publications Committee enjoyed, particularly since there was no longer any apparent reason for it as the circumstances that had engendered its independent authority had long ago been resolved. An appropriate revision was immediately drafted, and a handwritten copy delivered to Chuck Hancock, then the Executive Officer of the ASBMB, with instructions to bring this to Council for a vote as soon as it could be arranged. This was accomplished with dispatch and any crisis that was envisioned was averted.

Herb received many honors. He was given the Arthur S. Fleming Award in 1956; the USPHS Meritorious Service Medal in 1970; he was elected to the American Academy of Arts and Sciences in 1971, and to the National Academy of Sciences in 1977; he received the Hillebrand Prize from the American Chemical Society in 1986; he and Celia received the William C. Rose Award from the ASBMB in 1994. The ASBMB created an award in his name, The Herbert Tabor Research Award, presented annually for exceptional established investigators and the JBC created Herbert Tabor Early Career Investigator Awards in his honor. In 2018, the Montgomery County Council in Maryland designated November 28 as “Herb Tabor Day,” and the State of Maryland honored him with a ‘Governor’s citation’ in honor of his many contributions to science.

Those of us who have had the good fortune to work with Herb on editing the JBC and/or leading the Society will always remember him as a partner and friend who represented the very best of what the human race has to offer. He leaves a legacy of professional accomplishment in terms of scientific research and publishing that we can only marvel at. To say he will be missed is true but certainly not adequate; but there is also pleasure in knowing he will be fondly remembered, and hopefully emulated, by those who carry on his work (Figure 2).

**FIGURE 2:**
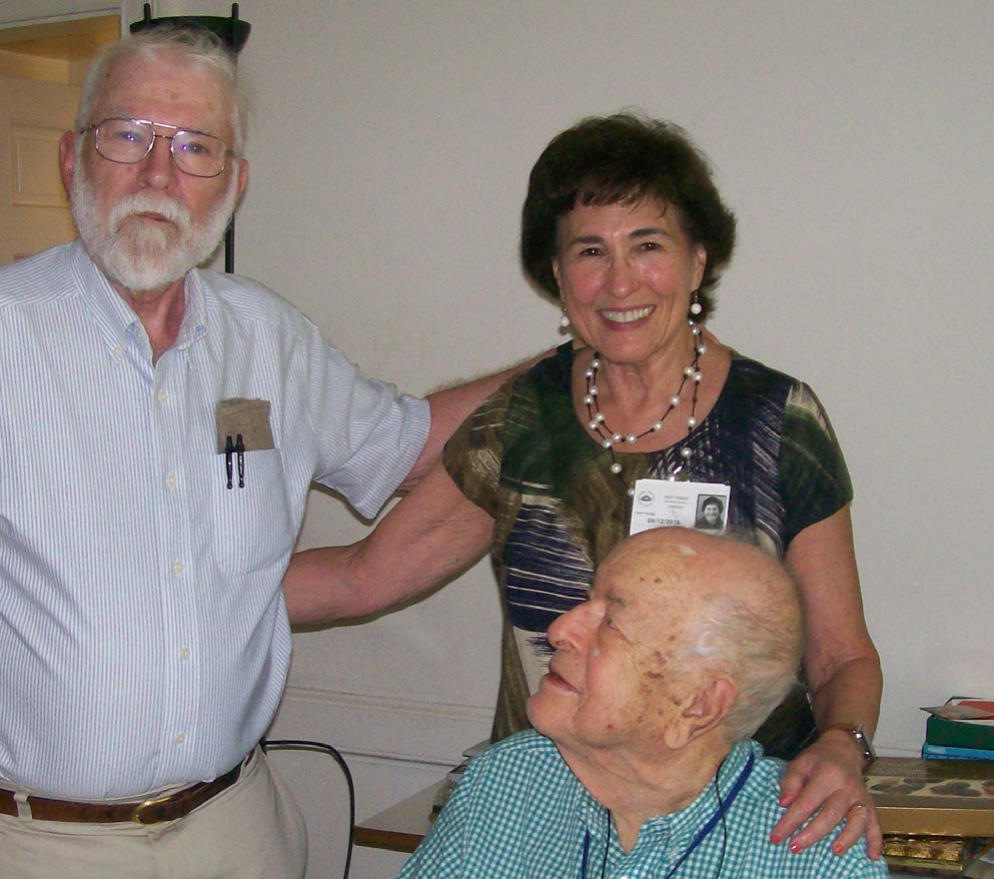
The authors (standing) with Herb at a lunch at his residence at the NIH, August, 2016 (photo by Gaylen Bradley)

## CONFLICT OF INTEREST

The authors have no conflict of interest.

